# Original Versus Generic Lenalidomide in Patients with Relapsed/Refractory Multiple Myeloma: Comparison of Efficacy and Adverse Events

**DOI:** 10.4274/tjh.galenos.2020.2020.0169

**Published:** 2021-02-25

**Authors:** Ali Zahit Bolaman, Atakan Turgutkaya, Birsen Sahip, Cem Selim, Hilal Eroğlu Küçükdiler, Şehmus Ertop, Gökhan Sargın, İrfan Yavaşoğlu

**Affiliations:** 1 Aydın Adnan Menderes University Faculty of Medicine, Department of Hematology, Aydın, Turkey; 2 Zonguldak Bülent Ecevit University Faculty of Medicine, Division of Hematology, Zonguldak, Turkey; 3 Aydın Adnan Menderes University Faculty of Medicine, Department of Immunology-Rheumatology, Aydın, Turkey

**Keywords:** Lenalidomide, Treatment, Original, Generic, Adverse effect

## Abstract

**Objective:**

Lenalidomide is an effective immunomodulatory derivative drug used in the treatment of multiple myeloma (MM). It is available in original and generic forms in Turkey, but there is no clinical study that has compared the effectiveness and adverse events (AEs) of the generic and original forms of lenalidomide. We compared the effectivity and AEs of generic and original lenalidomide in patients with relapsed/refractory MM (RRMM).

**Materials and Methods:**

Patients with RRMM using original or generic lenalidomide were evaluated retrospectively. Overall response (OR), complete response (CR), very good partial response (VGPR), partial response (PR), stable disease, and progressive disease rates and hematologic and nonhematologic AEs were evaluated in these RRMM patients. The results were described as numbers, frequencies, and percentages and were analyzed using PASW 19.0 for Windows with chi-square and Fisher exact tests.

**Results:**

The number of patients using original lenalidomide was 55 and the number of patients using generic lenalidomide was 43. The OR rate was 67.2% for patients using original lenalidomide and 60.4% for those on generic lenalidomide. CR and VGPR rates were 14.5% and 45.4% in the original group while the CR and VGPR rates were 20.9% and 18.6%, respectively, in patients using generic lenalidomide. Hematologic AEs were similar in the two groups while some nonhematologic AEs were less common in the original lenalidomide group than the generic group. Only pyrexia as a grade 3-4 AE was more common in the original lenalidomide than the generic lenalidomide group.

**Conclusion:**

This study showed that the generic form of lenalidomide has similar efficacy with the original form of lenalidomide in the treatment of RRMM. The AEs of original lenalidomide were generally fewer than those of generic lenalidomide. Further studies involving a larger number of patients with RRMM would be useful for comparing the efficacy and AEs of original and generic lenalidomide.

## Abstract (tr)

**Amaç:**

Lenalidomid, multipl myelom (MM) tedavisinde kullanılan, etkili bir immünmodülatuvar ilaçtır. Türkiye’de orijinal ve jenerik formları mevcuttur ve bu formların etkinliğini ve yan etkilerini karşılaştıran klinik çalışma yoktur. Bu çalışmada relaps/refrakter MM (RRMM) hastalarda jenerik ve orijinal lenalidomidin etkinliği ve yan etkileri karşılaştırılmıştır.

**Gereç ve Yöntemler:**

Orijinal veya jenerik lenalidomid kullanan RRMM hastaları, geriye dönük olarak değerlendirildi. RRMM hastalarında genel yanıt (OR), tam yanıt (CR), çok iyi kısmi yanıt (VGPR), kısmi yanıt (PR), stabil hastalık ve progresif hastalık oranları ve ayrıca hematolojik/hematolojik olmayan yan etkiler değerlendirildi. Sonuçlar, SPSS 19.0 programı yardımıyla, ki-kare ve Fisher testleri kullanılarak analiz edildi.

**Bulgular:**

Orijinal lenalidomid kullanan hasta sayısı 55, jenerik lenalidomid kullanan hasta sayısı 43 idi. OR oranı, orijinal lenalidomid kullananlarda %67,2, jenerik lenalidomid kullananlarda %60,4 idi. Orijinal grupta CR ve VGPR oranları %14,5 ve %45,4 iken, jenerik lenalidomid kullanan hastalarda CR ve VGPR oranları sırasıyla %20,9 ve %18,6 idi. Hematolojik yan etkiler her iki grupta da benzer iken, bazı hematolojik olmayan yan etkiler orijinal lenalidomid grubunda jenerik gruba göre daha düşüktü. Orijinal lenalidomid grubunda derece 3-4 yan etki olarak sadece ateş sıklığı, jenerik lenalidomid grubuna göre daha yüksekti.

**Sonuç:**

Bu çalışma, lenalidomidin jenerik formunun RRMM tedavisinde orijinal lenalidomid formu ile benzer etkinliğe sahip olduğunu göstermiştir. Orijinal lenalidomidin yan etkileri genel olarak jenerik lenalidomidden daha düşüktü. RRMM’li çok sayıda hastayı içeren daha ileri çalışmalar, bu iki formun etkinliğini ve yan etkilerini karşılaştırmak açısından yararlı olacaktır.

## Introduction

Multiple myeloma (MM) is an incurable disease that accounts for 1% of cancer across all age groups. The survival rate of patients has improved and long-term disease control has been achieved with the introduction of treatment strategies that consist of immunomodulatory drugs (thalidomide, lenalidomide, pomalidomide) and proteasome inhibitors (bortezomib, carfilzomib, and ixazomib) [1]. Lenalidomide is an oral immunomodulatory imide drug (IMiD) and an analogue of thalidomide. Lenalidomide is a tumoricidal drug because of its anti-proliferative, anti-angiogenic, and pro-apoptotic effects on plasma cells. It also increases T-cell-mediated and natural killer cell-mediated immunity, blocks pro-inflammatory cytokines (tumor necrosis factor, interleukin-6), and is effective on the bone marrow microenvironment in MM [2].

The antitumor effect increases synergistically if lenalidomide is used with dexamethasone. Lenalidomide plus dexamethasone (Rd) combination chemotherapy is an effective treatment option for both newly diagnosed and relapsed refractory MM (RRMM). The US Food and Drug Administration (FDA) initially approved IMiD compounds for RRMM based on the results from two clinical trials (MM-009 and MM-010) [3,4]. The Rd combination treatment was approved by the FDA based on phase 3 study results including the FIRST study (also known as MM-020 and IFM 07-01) for the first-line treatment of patients with newly diagnosed MM (NDMM) in 2015 [5]. The FDA also approved maintenance treatment with lenalidomide after stem cell transplantation in patients with MM in 2017 [6].

Lenalidomide has fewer adverse events (AEs) than thalidomide. The main AEs of lenalidomide were neutropenia, muscle cramps, constipation, nausea, tremor, and dizziness in the MM-009 and MM-10 studies [3,4,7]. Individual risk factors such as advanced age, a history of venous thromboembolism, an indwelling central venous catheter, comorbid conditions (infections, diabetes, cardiac disease, etc.), current or recent immobilization, and recent surgery can increase the AEs of lenalidomide therapy. The AE profile of lenalidomide is manageable and it has minimal cumulative toxicities, and the features of lenalidomide allow for long-term therapy. The risk of developing thrombosis and neuropathy is lower than that with thalidomide [8].

Original forms of lenalidomide have been used in all myeloma treatment studies reported so far. In Turkey, both the original and generic forms of lenalidomide can be used as first-line treatments as well as subsequent treatments of myeloma. There is no study that has compared the efficacy and the potential AEs of generic forms with the original form of lenalidomide. In this study, we report the efficacy and AEs of original and generic lenalidomide in RRMM patients who received these drugs as second-line treatment and who were not suitable for stem cell transplantation.

## Materials and Methods

A total of 98 patients with RRMM who were followed by the Division of Hematology at the Adnan Menderes University School of Medicine in Turkey between January 2014 and March 2019 were enrolled in this study. These patients with RRMM using original or generic lenalidomide were evaluated retrospectively. Patients who received at least two cycles of treatment were evaluated. The chemotherapy regimens were combination Rd regimens. Patients orally received 25 mg of lenalidomide for days 1-21 of a 28-day cycle and 40 mg of dexamethasone once a day every week [3]. The dexamethasone dose was reduced to 20 mg if the patient’s age was above 75. Acetylsalicylic acid (100 mg) was given to all patients undergoing lenalidomide treatment. Patients continued the Rd regimen until disease progression or unacceptable toxic effects were observed. Granulocyte colony-stimulating factor was applied if grade 3-4 neutropenia occurred and the lenalidomide dose was reduced for other AEs. Physical examination, blood count, and biochemical analysis were performed on days 1 and 15. Serum and urinary protein and immunofixation electrophoresis were evaluated every 3-4 cycles. Erythrocyte and platelet transfusions were given if needed and administration of neutrophil granulocyte factor was allowed when neutrophil count dropped below <500/µL.

Overall response (OR), complete response (CR), very good partial response (VGPR), partial response (PR), stable disease, and progressive disease rates were evaluated using the International Myeloma Group Criteria [9]. AEs including neutropenia, anemia, thrombocytopenia, febrile neutropenia, anorexia, constipation, diarrhea, nausea, vomiting, creatinine increase, transaminase increase, asthenia, fatigue, pyrexia, peripheral edema, upper respiratory system infection, pneumonia, other infections, muscle cramps, back pain, bone pain, muscle weakness, arthralgia, headache, tremor, paresthesia, deep vein thrombosis, pulmonary embolism, hyperglycemia, hypokalemia, hypocalcemia, hypomagnesemia, skin dryness, and skin erythema were investigated and graded according to the World Health Organization (WHO) Toxicity Scale and Cancer Therapy Evaluation Program [10,11].

### Statistical Analysis

All data were analyzed using PASW for Windows version 19.0 (SPSS Inc., Chicago, IL, USA). The results were described as numbers, frequencies, and percentages. The chi-square test and Fisher’s exact test were used for the analysis of categorical data and independence between variables. The Mann-Whitney U test was used to compare differences not normally distributed between groups. The results were assessed at a 95% confidence interval and p<0.05 was accepted as significant.

## Results

Of the patients involved in this study, 55 were using original lenalidomide and 43 were on generic lenalidomide. Previous treatments consisted of MPT or MPV regimens. The median follow-up was 11.2±10.3 months. There were no differences for age, sex, myeloma type, International Staging System (ISS) stages, follow-up time, ECOG performance status, previous therapy type, or β2 microglobulin (β2M) levels between patients receiving original and generic lenalidomide. The ISS stage could not be calculated for 31 patients (56.3%) in the original group and 20 patients (46.5%) in the generic group due to limitations of the β2M assay. The characteristics of patients receiving original and generic lenalidomide are shown in Table 1.

The OR rate was 67.7% (38 out of 55 patients) in the original lenalidomide group while it was 60.4% (17 out of 43 patients) in the generic lenalidomide group (Table 1). CR was observed in 8 patients (14.5%) in the original lenalidomide group and 9 patients (20.9%) in the generic group (p>0.05). The OR was higher but the CR rate was lower in the original lenalidomide group. VGPR was achieved in 25 (45.4%) patients and 8 patients (18.6%) in the original and generic groups, respectively (p=0.006). PR was achieved in 5 patients (7.2%) in the original group and 9 (20.9%) in the generic group (p=0.04). The progression rate was higher in patients in the generic group than the original group (4 versus 12 patients, p=0.006) (Table 2).

Neutropenia and thrombocytopenia were more common in the original lenalidomide group (61.8% versus 48.8% and 38.2% versus 34.9%, respectively), while anemia and febrile neutropenia were more common in the generic group (67.3% versus 76.7% and 40.0% versus 51.2%, respectively), although this was statistically insignificant irrespective of AE grade level. Nonhematologic AEs including nausea, vomiting, fatigue, edema, upper respiratory infection, pneumonia, back pain, pyrexia, muscle cramps and weakness, skin dryness, and skin erythema were more common in the generic group than the original group, and this was statistically significant (Table 3).

The most common hematologic grade 3-4 AE was neutropenia in both groups (20.0% versus 25.6%). Grade 3-4 nonhematologic AEs of more than 5% of patients were asthenia, pneumonia, and muscle weakness and these were more common in the generic group than the original lenalidomide group. Only grade 3-4 pyrexia was more common with statistical significance in the original lenalidomide group than the generic group (p=0.04).

Three patients (5.4%) in the original lenalidomide group and four patients (9.3%) in the generic group discontinued their drugs because of AEs. The causes of drug discontinuation were muscle weakness, pyrexia, and paresthesia in the original group and muscle weakness (two patients), headache, and insomnia in the generic group. We did not observe any fungal disease. There was only one viral disease (herpes zoster). The deep vein thrombosis rate was similar and we did not observe any pulmonary embolism in either group. There was no death due to AEs. No secondary malignancy was observed in either group during the follow-up. The lenalidomide dose was reduced for 7 of 55 (12.7%) patients receiving original lenalidomide and 6 of 43 (13.9%) patients receiving generic lenalidomide while dexamethasone was reduced for 16.3% and 23.2% of patients receiving original and generic lenalidomide, respectively.

## Discussion

IMiD drugs include thalidomide, lenalidomide, and pomalidomide. The first IMiD used in the treatment of myeloma was the parent drug thalidomide. Six randomized controlled trials compared melphalan and prednisone alone (MP) with a melphalan-prednisolone-thalidomide combination (MPT) for NDMM [12,13,14,15,16,17]. Fayers et al. [18] published a meta-analysis that included 1685 NDMM patients. Median progression-free survival was 14.9 months [95% confidence interval (CI): 14.0-16.6 months] with MP while it was 20.3 months with MPT. Median survival time with MP was 32.7 months (95% CI: 30.5-36.6 months), and with MPT, it was 39.3 months (95% CI: 35.6-44.6 months). Thalidomide has too many AEs in the treatment of myeloma. The rate of hematological AEs in MPT treatment is between 24% and 52%, and for nonhematological AEs the rate is between 12% and 23%. Thromboembolism (3%-12% versus 0%-4%) and neurological events (6%-23% versus 0%-4%) are the most common AEs in myeloma treatment with thalidomide. The addition of thalidomide to bortezomib-melphalan-prednisolone has increased treatment success but also the rate of hematological and nonhematological side effects in NDMM [19].

The efficacy and side effects of lenalidomide in Rd treatment without any third drugs such as melphalan or cyclophosphamide can be better evaluated. Adding a third drug to the Rd combination in the treatment of myeloma can cause difficulties when evaluating the effectiveness and AEs of lenalidomide. For example, there can be an increase in neurotoxicity as well as the effectiveness of the drug when bortezomib is added to the Rd combination [3,4,5,6]. AEs due to previously used chemotherapy or stem cell transplantation should also be taken into consideration in evaluating lenalidomide maintenance treatment [7].

OR rates of 61% and 60.2% were achieved in patients with RRMM in the MM-009 and MM-010 studies, respectively. Median progression-free survival was 4 months in both studies. While median OS was 29.6 months in MM-009, the median OS has not been reached yet in the MM-010 study. The most common hematologic grade 3-4 AE was neutropenia, at 41.3% and 29.5%, respectively, in the MM-009 and MM-010 studies [3,4]. The Rd combination formed the backbone of RRMM therapy in studies such as ASPIRE, POLLUX, TOURMALINE, PANORAMA-1, and ELOQUENT [20,21,22,23,24].

There are two forms of lenalidomide available in Turkey: original and generic. Ninety-eight patients with RRMM using original or generic lenalidomide were evaluated retrospectively in this study. The baseline characteristics of the patients did not differ between the groups (Table 1). The OR in our study with the Rd combination was similar in the original (67.2%) and generic lenalidomide (60.4%) groups and our results were similar to the results of MM-009 and MM-010. The CR rate was 20.9% in the generic lenalidomide group and 14.5% in the original lenalidomide group (p>0.5), whereas the VGPR rate was higher in the original lenalidomide group than the generic lenalidomide group, at 45.4% versus 18.6%, respectively (p=0.006). We do not know exactly why response rates were different between the groups. The OR rate in the previously received MPT regimen was 67.9% in the original group and 61.1% in the generic group. These rates in the previously received MPV regimen were 70.4% and 60% in the original and generic groups, respectively. We checked the β2M levels of only 47 of 98 patients and we found the OR rate in patients with β2M of ≥2.5 mg/L to be higher in the original lenalidomide group than the generic lenalidomide group (81.0% versus 47.4%, p=0.02) (Table 2). We did not evaluate survival analysis in our study.

Generally, grade 3-4 AEs rates were higher in the generic group than the original lenalidomide group. The most common grade 3-4 AE in the original lenalidomide group was asthenia (12.7% versus 4.6%), whereas in the generic group it was muscle weakness (11.6% versus 3.6%). Other grade 3-4 AEs of constipation and diarrhea, pyrexia, muscle cramps, and paresthesia were more common in the original lenalidomide group. However, grade 3-4 anorexia, nausea, vomiting, edema, upper respiratory infection, pneumonia, back pain, arthralgia, headache, insomnia, skin dryness, and erythema were more common in the generic group (Table 3). Grade 3-4 AEs between the groups were not statistically significant except pyrexia (p=0.04). There was no grade 3-4 deep vein thrombosis in either group. Dexamethasone was used by oral route at 40 mg on days 1 to 4, 9 to 12, and 17 to 20 for the first four cycles in the MM-009 and MM-010 studies [3,4]. We believe that the absence of grade 3-4 deep vein thrombosis and low rates of grade 3-4 pneumonia and grade 3-4 hyperglycemia may be related to dexamethasone use at 40 mg per week. The costs of generic and original lenalidomide are similar in Turkey. There are no studies in the literature comparing the effectivity and AEs of original and generic lenalidomide. To the best of our knowledge, this paper is the first study to compare original and generic lenalidomide. In our study, the response rates and the results obtained in RRMM patients using original and generic lenalidomide were similar. We observed some differences in OR and CR (nonsignificant) and VGPR and PR (significant at p=0.0006 and 0.04, respectively). AE rates were significantly higher in the generic group than the original group, but only pyrexia was of grade 3-4. We believe this may be associated with the small number of patients.

### Study Limitations

The study has some limitations. One is that the number of patients in the study was limited and we were unable to evaluate β2M levels for some patients. Since original versus generic lenalidomide use is not the only factor in prognosis and ISS is known to be one, further determination of patients with unknown stages would contribute to more accurate prognostic information. Furthermore, the OR, CR, and VGPR rates were higher in patients with β2M of ≥2.5 mg/L (p=0.02) in the original generic group (p=0.05); higher VGPR and PR rates (p=0.006 and p=0.04, respectively) in the generic group may also be associated with the low number of patients. Further studies involving more patients are needed to explain why there were different response rates regarding original versus generic lenalidomide.

## Conclusion

This retrospective study has showed that the overall response and AE rates of original and generic lenalidomide are similar to each other. Further research involving more patients would be useful to compare the response rates and AEs of original and generic lenalidomide.

## Figures and Tables

**Table 1:**
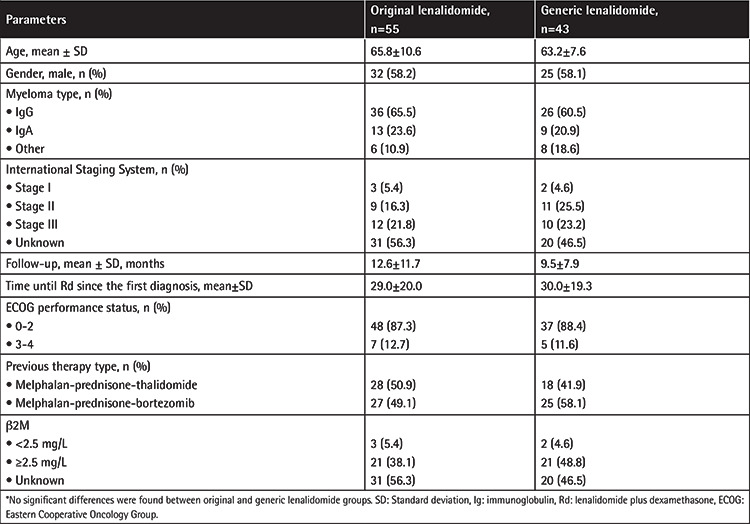
Baseline characteristics of relapsed/refractory multiple myeloma patients using original and generic lenalidomide.*

**Table 2:**
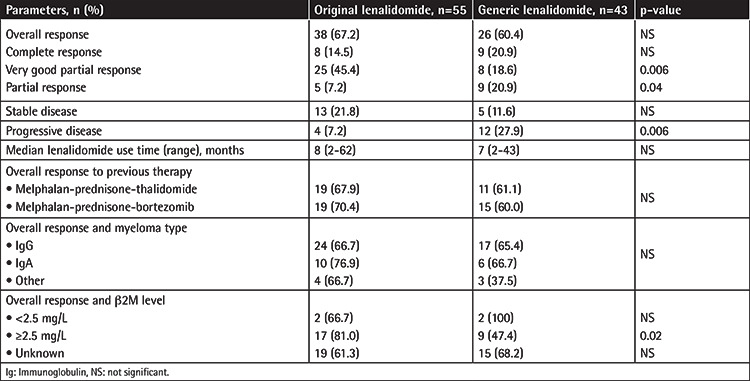
Response of relapsed/refractory multiple myeloma patients receiving original and generic lenalidomide.

**Table 3:**
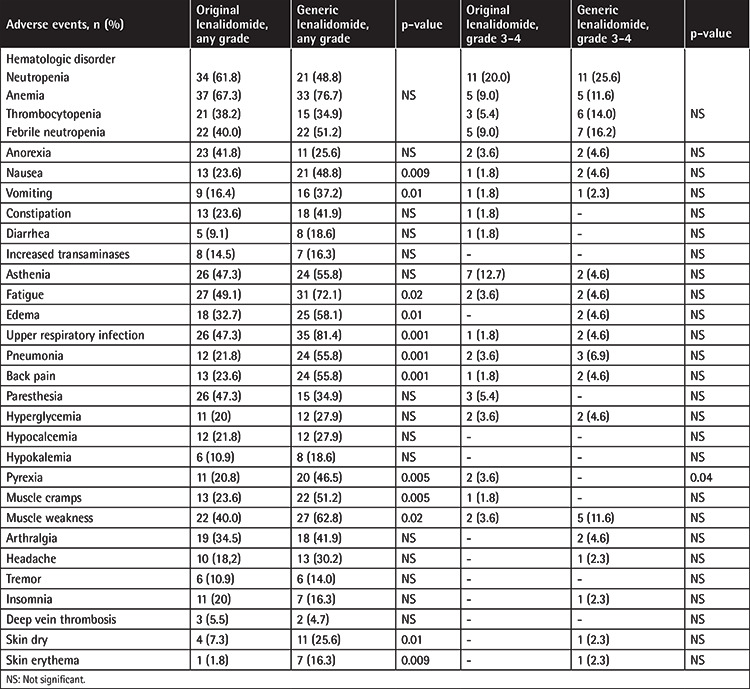
Distribution of adverse event grades in relapsed/refractory multiple myeloma patients receiving original or generic lenalidomide.
